# Strategies for Restoring and Managing Ecological Corridors of Freshwater Ecosystem

**DOI:** 10.3390/ijerph192315921

**Published:** 2022-11-29

**Authors:** Qiaoyan Lin, Yu Song, Yixin Zhang, Jian Li Hao, Zhijie Wu

**Affiliations:** 1 The XIPU Institution, Xi’an Jiaotong-Liverpool University, Suzhou 215123, China; 2 Department of China Studies, Xi’an Jiaotong-Liverpool University, Suzhou 215123, China; 3 Department of Landscape Architecture, Gold Mantis School of Architecture, Soochow University, Suzhou 215123, China; 4 Department of Civil Engineering, Design School, Xi’an Jiaotong-Liverpool University, Suzhou 215123, China; 5 Research Institute for Environmental Innovation (Suzhou), Tsinghua, RIET, Suzhou 215163, China

**Keywords:** urbanization, habitat degradation, ecosystem restoration, ecological corridor, ecological network

## Abstract

Along with accelerating urbanization and associated anthropogenic disturbance, the structure and function of freshwater ecosystems worldwide are substantially damaged. To improve ecosystem health, and thus enhance the ecosystem security of the urban ecosystem, numbers of management approaches and engineering projects have been applied to mitigate the degradation of freshwaters. Nevertheless, there is still a lack of comprehensive and systematic research on the ecological corridor restoration of freshwater ecosystems; especially for Suzhou Grand Canal, one section of the world’s longest and ancient Grand Canal which is inclined to severe ecosystem degradation. Through investigating the adjacent land use characteristics, habitat quality, vegetation cover, instream water quality, and habitat composition, we aimed to: (i) assess the water quality of the Suzhou Grand Canal; (ii) evaluate the ecological characteristics of the canal ecosystem; (iii) develop strategic countermeasures to restore the ecological corridors for the mitigation of ecological problems. The results demonstrated: a large built area, a smaller ecological zone, a low habitat quality and habitat connectivity, and a high degree of habitat fragmentation within the canal corridor, also a simplified instream habitat composition, and greater nutrient and COD concentrations in the surface water—especially in the upstream and midstream canal. All urbanization-induced multiple stressors, such as land use changes, altered hydrology, and the simplified riparian zone et al., contributed synergistically to the degradation of the canal ecosystem. To alleviate the ecosystem deterioration, three aspects of recommendations were proposed: water pollution control, watershed ecosystem restoration, and ecological network construction. Basically, building a comprehensive watershed ecological network—on the basis of associated ecosystem restoration, and the connection of multi-dimensional ecological corridors—would dramatically increase the maintenance of aquatic–terrestrial system biodiversity, and improve the regional ecological security pattern and watershed resilience toward stochastic future disturbances. This study contributes to the understanding of the ecological challenges and related causes of the canal ecosystem. The integrated strategy introduced in this study provides policymakers, water resource managers, and planners with comprehensive guidelines to restore and manage the ecological corridor of the canal ecosystem. This can be used as a reference in freshwater ecosystems elsewhere, to improve ecosystem stability for supporting the sustainable development of urban ecosystems.

## 1. Introduction

Serving as the main linkage between terrestrial and aquatic habitats for the concern of species ecology (e.g., the requirement of living history, preferred niche, dispersal abilities) [1], freshwater ecosystems enable the multidimensional dispersal of organisms [2] and play important roles in the cycling of matter, and flow of energy [3,4]. For instance, ecological continuity not only facilitates sediment transport, nutrient cycling, and energy flow, but also allows the spread of aquatic invertebrates, and fish in rivers and streams [5,6]. However, under challenges of global climate change and urbanization-induced human disturbance, riverine ecosystems are ranked as the most diverse and threatened ecosystem in the world [7]. With hydro-morphological and physico-chemical changes, most rivers, both in sparsely populated areas [8] and surrounded by densely populated regions, are inclined to habitat deterioration and river corridor disruption [9,10]. Habitat loss, fragmentation, and corridor interruption negatively impact on aquatic biodiversity, ecosystem structure, ecosystem function, ecosystem processes and services of freshwaters [11,12,13,14], which in turn influence the health of human beings [15,16]. Moreover, the intense urbanization causes an increasing contradiction between socio-economic development and ecological conservation [17].

With the progress of habitat fragmentation/loss in freshwaters worldwide, much effort has been put into ecological restoration of riverine habitats and their connectivity [2,18,19]. For instance, approaches including the removal and modification of dams, stream-crossings, or construction of fish passages, were applied to restore the longitude connectivity, which helps to restore natural riverine processes thus creating living habitats for aquatic biotas [20,21]. Laterally, restoring connections between the main channel and its floodplain, wetlands support the recovery of riverine functions, such as the natural exchange of nutrients between the floodplain and mainstream, and the development of a greater diversity of riparian conditions and habitat types [20,22]. As “a linear two-dimensional landscape element that connects two or more patches of wildlife (animal) habitat” [23], an ecological corridor has been proposed as one way to stimulate the formation of a more complete ecological network [17], and to enhance the persistence, transmission, and dispersal of water-dependent species which could maintain key ecological processes [24,25]. A river ecological corridor includes the meander belt of a stream and a buffer of 50 feet [26]; it enforces the connection and interaction of core river and adjacent rivers, grasslands, parks, wetlands, or lakes, to form an integrative, stable, and equilibrium river ecosystem [27,28]. Healthy river corridors play key roles in ecosystem function, such as through the filtration of sediment and pollutants [29], ecological landscape construction [17], and provide important spatial context for maintaining the corridor species, and sustaining ecosystem processes and services for human beings [6,30]. Thereby, rivers in good condition have the ability to maintain sustainable development under anthropogenic disturbance, climate change, and climate change-related catastrophic flood damages. However, most river corridors are highly human-modified, or deteriorate at an increasing pace [31,32].

As an emerging concept of systematic watershed restoration, research on river corridor protection and restoration is growing. River corridors are purposely restored to protect specific landscape elements, and facilitate landscape connectivity for regional species, ecological communities, or sustainable flood protection [33,34]. Based on scientific evidence, river ecological corridor management is a critical measure to recover the freshwater ecosystem and achieve harmonious coexistence between the ecological environment and human beings [35]. Research proved that river corridor restoration approaches have been quite useful in alleviating negative flood impacts, while improving the ecological viability and health of degraded freshwaters [36,37]. Ecological corridor management has also been likely to protect, or improve, movement and gene flow for flora and fauna inhabiting designated ecological corridors [38]. Moreover, restoration of river ecological corridors can reduce the adverse effects of habitat fragmentation caused by rapid urban development, which is crucial to enhance the linkage of ecological patches, and recover the ecosystem function and service [39]. Among numerous studies accumulated, most studies have been mainly focused on the restoration and management of specific components of river corridors, either on riparian corridors [40,41], or overall habitat connectivity [42,43], since riparian corridors support greater biodiversity and ecological processes than other landscape elements [44]. Studies concerning the whole catchment area—both instream habitat connectivity and quality, as well as riparian vegetation and land use forms—were scarce [14]. Some studies summarized a series of ecological protection and restoration measures and technologies that are adequate for different degradation situations in various regions [38,45,46]. However, there is still a lack of research on the restoration of river ecological corridors as a system. Furthermore, little comprehensive research has been conducted on the ecological protection and corridor restoration of the Grand Canal, a man-made ancient freshwater ecosystem which experienced remarkable hydro-morphological changes under speeding urbanization. Therefore, the countermeasures of ecological corridor restoration and management need to be further strengthened and enriched.

Suzhou Grand Canal is one section of the Beijing–Hangzhou Grand Canal constructed thousands of years ago. During the past few decades, it has experienced remarkable changes in land use cover, under progressively intensified anthropogenic disturbance. The canal was channelized with a concrete riverbank, and enriched with heavy loads of pollutants, which engendered a negative impact on the aquatic environment and canal ecosystems. Furthermore, with the speeding urbanization and urban renovation, against the background strategy of Yangtze River Delta Integration, a decrease in natural land with high habitat quality and an increase in construction lands, were detected [47]; undoubtedly, the contradiction between socio-economic development and ecological conservation is increasing [17]. The Suzhou Grand Canal is the ideal study area to establish ecological corridors and an ecological network for the sustainable development of Suzhou city. Hence, we have taken the Suzhou Grand Canal as a case study. Through investigating the adjacent land use characteristics, riparian habitat quality, vegetation coverage, instream water quality, and habitat composition, the objectives of this study are to: (i) investigate the water quality of the Suzhou Grand Canal; (ii) evaluate the ecological status of the canal ecosystem; (iii) develop strategic countermeasures to restore ecological corridors for the mitigation of ecological problems. Based on ecological theory and scientific evidence, that are likely to help conserve or restore the integrity of the freshwater ecosystem, we put forward our recommendations on how to develop a systematic strategy to construct a systematic connected ecological corridor for the freshwater ecosystem. Practically, this study will contribute to the understanding of the ecological problems and influential factors of freshwaters, and will help policymakers and water resource managers to work out a comprehensive strategy for the restoration and management of the degraded freshwater ecosystem.

## 2. Materials and Methods

### 2.1. Study Area

**Figure 1:**
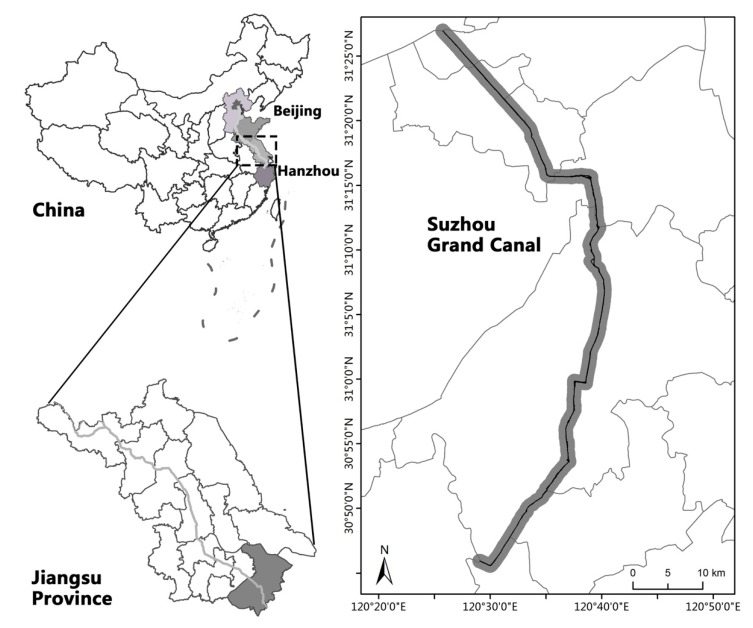
Study area of the Suzhou Grand Canal within Suzhou City Region, PRC.

Suzhou Grand Canal is one section of the Beijing–Hangzhou Grand Canal located within the Suzhou city (120°44′ E–120°60′ E, 30°86′ N–31°45′ N), China (Figure 1). Situated at the Tai Lake Basin in the downstream of Yangtze River, it starts from Wangting town in Xiangcheng district, and ends in Taoyuan town in Wujiang district (Figure 1). It accounts for 4.5% of the Beijing–Hangzhou Grand Canal; the canal is about 96 km long, 20 m wide, and 2 m deep, with an average annual runoff of 58 × 108 m^3^ and an average annual precipitation of 1048 to 1094 mm [48]. With more than 6000 navigable vessels passing through the canal every day, the annual navigation volume of the Suzhou Grand Canal accounts for one-fifth of the total navigation of the Grand Canal, which makes the canal one of the busiest sections of the entire Beijing–Hangzhou Grand Canal. Suzhou Grand Canal is also one of the world’s cultural heritages, and it contains four canal-related heritage sites and three canal hydraulic relics.

Present as the lateral distance from the centerline of the river to both sides of valley walls, the width of the ecological corridor determines the ecological structure and function of the freshwater ecosystem [49,50,51]. As Jiang et al. (2016) and Tzolova (1995) suggested, the optimal width of an urban stream corridor and its related functional region ought to be approximately 1000–3000 m, from the perspective of ecosystem structure and function [52,53]. Xiao et al. (2020) proposed that most terrestrial species in Jiangsu Province disperse from 3 to 2000 [54]. Accordingly, we set ecological conservation as the major determinant of the canal ecological corridor. By combining the land use cover along the Suzhou Grand Canal with the “Detailed rules for the management and control of land and space in the core monitoring area of the Grand Canal in Suzhou”, the ecological corridor width of the canal is defined to 2000 m, and the study area is around 186.32 km^2^. To investigate the ecological status of different regions, we denoted the study areas in Xiangchen and Gaoxin districts as upstream canal, the study areas in Gusu and Wuzhong districts as midstream canal, and the study areas in Wujiang district as downstream canal.

### 2.2. Data

The land use/land cover (LULC) data with a spatial resolution of 10 m was collected from Esri Global Land Cover Map. This map was derived from European Space Agency (ESA) Sentinel-2 imagery and was a composite of LULC predictions for each year from 2017–2021 [55]. One cloud-free Sentinel-2 image which covered the study area, was collected on 12 July 2021 from Copernicus Open Access Hub (https://scihub.copernicus.eu/). We adopted the multispectral image to extract the water boundary. The land use type was classified into six categories, including built area (residential land, commercial land, industrial land, mining land, and transportation land), cropland (paddy field, cropland), forest land (forest land, forest swamp, and other forest land), grassland (high coverage grassland), wetland (rivers, lakes, shallows, beach, and swamps), and bare ground (bare land, free area). The total area of built area, cropland, forest land, grassland, wetland, and bare ground in the upstream, midstream, and downstream canal were then calculated, respectively. The LULC data in 2021 has been adopted to assess habitat quality, and habitat degradation.

To supplement the land use situation along the canal, the number of manufacturing industries, agricultural companies, and Sewage Treatment Plant surrounded by the Suzhou Grand Canal, were then calculated using the Qichacha database as supporting information.

### 2.3. Habitat Quality and Habitat Degradation

Habitat quality reflects the suitability of a habitat land for biodiversity conservation [56]. Habitat degradation reveals the intensity of threats on habitat [57]. Both habitat metrics range between 0 and 1; a lesser score of habitat quality indicates a habitat with a lower adequacy for the living of species, and a greater habitat degradation score implies a lower habitat quality [56]. Both habitat metrics were calculated by InVEST Habitat Quality model [58,59]. The model combines maps of LULC, with data on threats to habitats and habitat response. This approach generated two key sets of information that are useful in making an initial assessment of conservation needs: the relative extent and degradation of different habitat types in the study region.

We ran the model using raster data, where each cell in the raster was assigned to a LULC class. Wetland, forest land, grassland and cropland were considered as habitats in this study. Additionally, a threats table was generated to map each threat of interest to its properties and distribution maps. Table 1 shows the maximum distance over which each threat affects habitat quality, the impact of each threat on habitat quality, and the type of decay over space for each threat (effects of the threat decay exponentially or linearly with regards to distance from the threat).

The impact of threat *r* that originates in grid cell *y*, *r_y_* on habitat in grid cell *x* is given by *i_rxy_* and is represented by the following equations:

(1) $$i_{rxy}=1-\left(\frac{d_{xy}}{d_{r\;max}}\right)\;\mathrm{if}\;\mathrm{linear}$$

(2) $$i_{rxy}=exp\left(-\left(\frac{2.99}{d_{r\;max}}\right)d_{xy}\right)\;\mathrm{if}\;\mathrm{exponential}$$

where *d_xy_* is the distance between grid cells *x* and *y*, *d_rmax_* is the maximum effective distance of threat *r*’s reach across space.

In addition, the model also developed a sensitivity table which maps each LULC class to the species’ habitat preference (where 0 is not suitable and 1 is completely suitable) and threat sensitivity (where 1 represents high sensitivity and 0 represents that it is unaffected) in the corresponding areas (Table 2).

The total threat level in grid cell *x* with LULC or habitat type *j* is given by *D_xj_*.

(3) $$D_{xy}=\Sigma_{r=1}^{R}\Sigma_{y=1}^{Y_{r}}\left(\frac{w_{r}}{\sum_{r=1}^{R}w_{r}}\right)r_{y}i_{rxy}\beta_{x}S_{jr}$$

where *y* indexes all grid cells on threat *r*’s raster map and *Y_r_* indicates the set of grid cells on *r*’s raster map. *β_x_* indicates the level of accessibility in grid cell *x* where 1 indicates complete accessibility. *S_jr_* indicates the sensitivity of LULC to threat *r* where values closer to 1 indicate greater sensitivity.

A grid cell’s degradation score was translated into a habitat quality value using a half saturation function:

(4) $$Q_{xj}=H_{j}\left(1-\left(\frac{D_{xj}^{z}}{D_{xj}^{z}+k^{z}}\right)\right)$$

where *Q_xj_* is the habitat quality in parcel *x* that is in LULC type *j*. The *z* and *k* are scaling parameters. *H_j_* is the habitat suitability of LULC type *j*.

### 2.4. Habitat Fragmentation and Habitat Connectivity

To measure the landscape patterns, wetland, forest land, grassland and cropland were used as habitats, to analyze landscape metrics (habitat fragmentation and habitat connectivity) in this study. Two class-level landscape metrics were introduced, for calculation with Fragstats 4.2 (University of Massachusetts Amherst, Massachusetts, United States of America; a computer software program designed to compute a wide variety of landscape metrics for categorical map patterns). Habitat fragmentation and habitat connectivity index were expressed in equations as follows.

(5) $$FD=n/A_{i}$$

where *FD* is the habitat fragmentation index. *n* is the number of patches. *A_i_* is the total area of the patch in hectares. *FD* increases as the patch type is increasingly fragmented.

(6) $$CONNECT=\left[\frac{\sum_{j=k}^{n}C_{ijk}}{\frac{n_{i}\left(n_{i}-1\right)}{2}}\right](100)$$

where *CONNECT* is the habitat connectivity index; *C_ijk_* was joining between patch *j* and *k* (0 = unjoined, 1 = joined) of the class level landscape (*i*), based on a user-specified threshold distance. *n_i_* is the number of patches in the class level landscape. *CONNECT* equals 0 when either the focal class consists of a single patch, or none of the patches of the focal class are connected. *CONNECT* equals 100 when every patch of the focal class is connected.

### 2.5. Vegetation Coverage

The surface reflectance values of B4 and B8 in Sentinel-2 image were used to calculate the Normalized Difference Vegetation Index (NDVI). Referring to the China Technical Criterion for Ecosystem Status Evaluation (HJ 192-2015), the percentage of vegetation coverage (PVC) was measured in an equation as follows:

(7) $$NDVI=\left(B8-B4\right)/\left(B8+B4\right)$$

(8) $$PVC=A_{veg}\times \frac{\sum_{i=1}^{n}P_{i}}{n}$$

where *A_veg_* is the conversion coefficient (0.0121165124). *P_i_* is the NDVI value. *n* is the number of pixels. The PVC equals 0 when the cell is bare soil, and equals 1 when the cell is fully covered by vegetation.

### 2.6. Water Quality and Instream Habitat Characteristics

Physico-chemical characteristics of surface water were measured in two or three sampling spots, in five sections of the canal. A 200 milliliter water sample was randomly collected from each spot, stored at −4 °C, and analyzed within 48 h for a range of chemical measures, including pH, dissolved oxygen (DO), ammonium nitrogen (NH_4_-N), nitrate nitrogen (NO_3_-N), total nitrogen (TN), total phosphorus (TP) [60], and chemical oxygen demand (COD) [61]. All physio-chemical parameters in the five sections were then compared to the standard level IV water quality, to evaluate the current aquatic environment in the canal (Table A1). The TN and TP were used as nutrient indicators to evaluate the nutrition status of the canal, according to Criteria and Grading Methods for Nutritional Status Evaluation in the Technical Regulations for Quality Evaluation of Surface Water Resources SL 395-2007 (Table A2). At each sampling spot, the instream habitat composition was then visually estimated by counting the riverbed types including riffle, pool, and island, within a 50 m sampling reach.

## 3. Results

### 3.1. Land Use Characteristic

**Figure 2:**
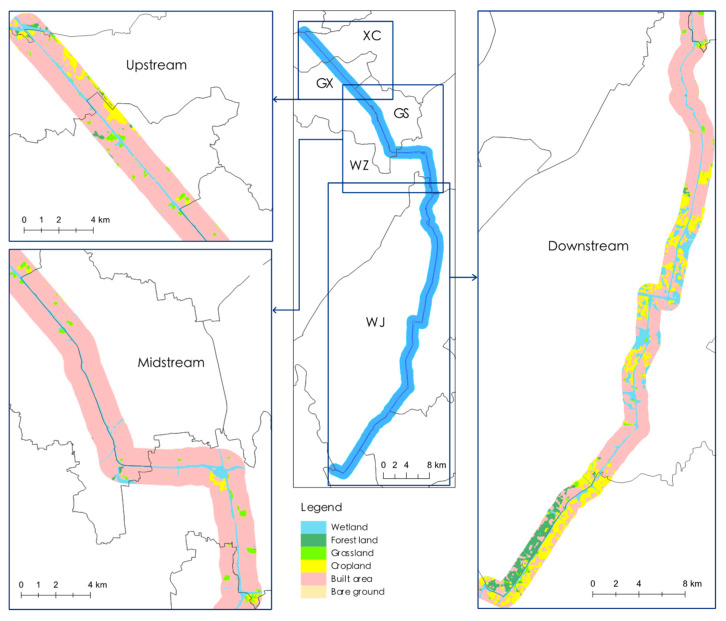
Land use types of adjacent Suzhou Grand Canal in 2021.

As illustrated in Figure 2, the land use types along the Suzhou Grand Canal are relatively rich. Built areas including industrial parks, factories, commercial areas, and residential areas occupied most of the study area, followed by ecological assets such as wetland, cropland, forest land, grassland, and other land, such as bare ground. All components are interconnected, forming an urbanized watershed ecosystem linked either by green vegetation belts or man-made infrastructures.

The upstream canal involved 84.52% of built area, 6.39% of wetland, and 9.09% of other areas comprising cropland, forest land, and grassland. The midstream canal was also rich in built area (87.28%) and wetland (9.27%). The downstream canal, however, was mostly occupied by ecological resources (including 15.21% of wetland, 13.71% cropland, 6.98% of forest land, and 1.446% of grassland), except for a large proportion of built area (62.63%).

### 3.2. Potential Risks

Except for widespread cropland distributed in the canal region, the built area also contained large numbers of industrial parks, which may cause potential risks to the entire canal watershed ecosystem. According to the survey (Table 3), the built area around the upstream canal contained 123 manufacturing factories that dispersed in large numbers of industrial parks. The midstream region had the greatest percentage of built area (87.28%), which was surrounded by industrial parks comprising 131 industrial companies. The downstream canal covered 66.26 km^2^ of built area and 14.50 km^2^ of cropland, surrounded by 2539 manufacturing industries in chemistry, textile, papermaking, concrete, and metallurgy.

Above all, although the downstream canal had a larger area of cropland, and a large amount of manufacturing industries that have high potential risks to aquatic environment, it also contained a larger area of ecological resources and wastewater treatment plants (WWTPs), than other parts of the canal. The greatest residential areas were detected in the midstream canal, among all canal regions.

### 3.3. Habitat Feature

**Figure 3:**
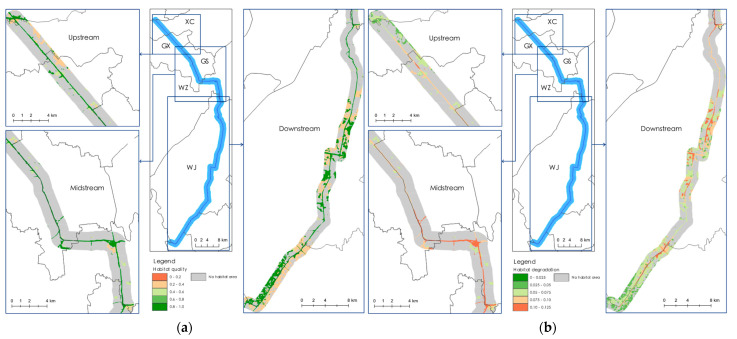
Spatial distribution of habitat quality (**a**) and habitat degradation (**b**) in the Suzhou Grand Canal in 2021.

In view of the instream habitat composition, almost all sections of the core river channel were constituted by pools; only small amounts of islands and floodplains were embellished in the downstream and midstream canal. No markable difference of the instream habitat composition was observed among three sections of the Suzhou Grand Canal. With regard to canal corridor, the habitat quality score was less than 0.3 in all regions studied. With greater amounts of ecological resources surrounded by the downstream canal, the mean habitat quality was greatest in downstream, followed by canal sections in upstream and midstream (Figure 3, Table 4). In addition, the habitat degradation showed a greater habitat degeneration rate in midstream, and a lesser degradation rate in upstream. The vegetation coverage within the study area was greatest in downstream, and lowest in midstream (Table 4).

The mean habitat connectivity of the canal was greater in midstream, followed by that in upstream and downstream. In terms of habitat fragmentation, the habitat in upstream was much more fragmented than in other sections of the canal; the downstream canal, however, experienced the least fragmentation among the study area (Table 4).

### 3.4. Physico-Chemical Index

No difference in physico-chemical index was detected among all sections of the canal with regard to NH_4_-N and TP (*p* > 0.05), whereas physico-chemical indexes including pH (F = 8.750, *p* = 0.007), DO (F = 71.710, *p* = 0.000), NO_3_-N (F = 107.350, *p* = 0.000), TN (F = 11.184, *p* = 0.004), COD (F = 8.205, *p* = 0.009), and conductivity (F = 17.743, *p* = 0.001) were significantly different among various canal sections. River sections (D and E) in the upstream and midstream canal had a greater pH, NO_3_-N, TN, COD, conductivity, and a lower concentration of DO than the surface water in downstream (Table 5). The water quality in downstream was much better than that in the midstream and upstream canal.

When comparing water quality in the Suzhou Grand Canal with standard class IV water, most variables tested were lower, or complied with the class IV water, according to the Surface Water Environmental Quality Standard GB3838-2002. The exception was a much higher concentration of TN, NO_3_-N, and COD in the surface water of the midstream canal (Table 5 and Table A1). TN and TP concentration were lowest in the downstream canal, whereas the nutrient concentration tested in all sections exceeded the standard nutrition grade. According to Criteria and Grading Methods for Nutritional Status Evaluation in Technical Regulations for Quality Evaluation of Surface Water Resources SL 395-2007, the overloading of nutrients had caused the middle eutrophication of the grand canal (Table A2).

## 4. Discussion

### 4.1. Aquatic Environment—Status, Causes, and Recommendations

#### 4.1.1. Water Quality

The Suzhou Grand Canal is surrounded by fast-developed built area, with dense industries and residential areas, wide distribution of agricultural lands, and ecological resources including wetland, forest land, and grassland, resulting in different degrees of pollution (e.g., industrial pollution, agricultural pollution, domestic sewage pollution, etc.) in most of the river sections monitored. Since 2007, lots of management projects were applied to restore and protect the instream and riparian zone environments [62], and the water quality of the canal has been upgraded from Class V to Class IV. However, the surface water still possessed a greater concentration of nutrients (e.g., NO_3_-N, TN, TP). The overload of nutrients led to middle eutrophication of the river, which may cause adverse impacts on aquatic organisms, the canal ecosystem, and human health [63]. It is still challenging to protect the aquatic environment of the canal freshwater ecosystem.

River eutrophication may come from a variety of resources, from tail water discharged from wastewater treatment plants, runoff or leaching from construction lands in urban areas, or agricultural lands in suburbs [64,65]. Based on the land use types adjacent to Suzhou Grand Canal, excessive nutrients generated from residential discharge, run off from built areas, and the agriculture backwater enriched with fertilizer, might mainly contribute to the eutrophication of the canal—especially for water in the upstream and midstream canal. This finding is consistent with the research by Matta (2017), who indicated that discharge of sewage, industrial effluent, and commercial and domestic waste into the canal, was responsible for the high organic and nutrient pollution [66]. Additionally, since tail water from wastewater treatment plants possesses greater nutrients, such as total nitrogen and total phosphorus [67], the tail water discharged into the canal or tributaries might be another cause of eutrophication.

#### 4.1.2. Recommendations for Aquatic Environment Protection

Aquatic environmental problems, including river eutrophication, can not only lead to cyanobacterial blooms and depletion of dissolved oxygen in the water [61,68]; major changes in aquatic communities [69,70]; degradation of ecosystem processes and functioning by altering interspecific relationships in food webs [71,72]; but may also pose serious threats to the urban ecosystem, and our human health [63,73]. The data concluded that exogenous contaminants are significant contributors to the deteriorating environmental health of the canal. Identifying pollution sources and developing mitigation plans would significantly address the problem; thus, aiding the recovery of the aquatic environment to a healthy status to sustain diverse aquatic communities, and community related ecosystem process and function. Firstly, due to interconnection with a wide distribution of river tributaries and lakes, it is critically important to identify the exogenous pollution through regular monitoring and assessment. The pollution sources could be actively controlled by strengthening the interception of exogenous contaminants, in situ treatment of sewage and sediment, and ecological restoration of freshwater ecosystems [74,75,76].

Secondly, for water pollution caused by adjacent industries, agricultures, domestic residents, and tailwater from wastewater treatment plants, approaches based on process-based principles could be coordinatively taken to prevent and control water pollution by addressing the causes of degradation [8,77,78]. Green Productivity is a strategy for enhancing productivity and environmental performance simultaneously [79]. The point source of pollution could be drastically mitigated at its source through promoting Green Productivity via industrial transformation and upgrading, agricultural ecological transformation, sponge city transformation, as well as improvement of sewage treatment skills and industrial discharge standards [80,81,82]. Additionally, runoff attenuation features (RAFs) (e.g., buffer belts, shelter forests, wetland systems, etc.) could be constructed by the pollution source (e.g., built areas or croplands) [14]. This is because RAFs can be used to reduce runoff-associated non-point sources of pollution, through the slowing, filtering, and infiltration of runoff pollutants [74]. Moreover, some aquatic plants (e.g., Typha latifolia, Phragmites australis) and microorganisms can actively transform/mobilize nutrients from contaminated water [83,84]. The re-introduction and management of aquatic plants and microorganisms/biofilms in the aquatic system, could be adopted to remove excessive nutrients.

### 4.2. Freshwater Ecosystem

#### 4.2.1. Ecological Status of the Canal Ecosystem

Healthy freshwater ecosystems consisting of heterogenous riverine habitats, not only support the persistence of aquatic biota (e.g., aquatic flora, fauna, micro-organisms), but also provide essential ecosystem services for human beings, for example by reducing pollutants, providing water/food for human life [85,86]. However, land use changes and anthropogenic activities caused by fast urbanization, have significantly changed the habitat characteristics, altered the composition of biological communities, and shifted the biogeochemical processes of various water bodies [84,87]. With speeding urbanization, the built area was increased, the floodplain and riparian zones were simplified, and the ecological resources (e.g., forest land, grassland, and wetland) of the canal system were shrunk. These changes led to the simplification of habitat composition, the degradation of habitat quality in the canal corridor, as well as the degradation of the canal ecosystem in this study.

Multiple stressors caused by urbanization have been attributed to the destruction of freshwater ecosystems, for instance through land use change, water withdrawal and pollution, damming, and invasive species [88]. Particularly, the freshwaters of China are at risk because of a variety of conflicts surrounding water use in urbanization, industry, and agriculture [12]. Here in this study, although 153 km of embankment reinforcement project was conducted in the recent few years to enhance the flood control, lots of environmental remediation projects were implemented to improve the canal environment [89]. The habitat condition is not compatible for the construction of a highly interconnected ecological network. It is still challenging to restore the integrity and stability of the canal freshwater ecosystem.

#### 4.2.2. Recommendations for Ecosystem Restoration

Without establishing an integrative and stable ecosystem, it is difficult to maintain valuable ecosystem service, as well as the ecological security pattern for human beings [90]. Hence, there is an urgent need to restore the structure and functioning of freshwater ecosystem, such as the canal. In view of the degradation of habitat quality and the canal ecosystem, applying regional habitat restoration and systematic ecosystem restoration would substantially promote the recovery of ecosystem structure and function, within the ecological corridor.

Firstly, riverine habitat components such as floodplains, riverine wetlands, as well as surrounding tributaries and wetlands, are fundamental in order to sustain the living history of various biological organisms, and to recover the integrity of the ecosystem structure [91,92]. Developing restoration and conservation approaches to restore the riverine habitat in regions (e.g., downstream canal in this study) that had greater habitat degradation, would improve the survival of diverse aquatic biota and organism-related ecosystem functions. Riverine habitat could be remarkedly restored through floodplain rehabilitation, re-introduction of aquatic plants, and the increase of habitat heterogeneity [84]. Furthermore, riverbank and riparian zones are two key areas that possess aquatic–terrestrial habitats to sustain the aquatic–terrestrial biota [92,93]. Enhancing the integrity of the riparian ecosystem through ecological riverbank protection, river–lake wetlands connection, riparian reforestation, etc., would improve the spatial structure for aquatic–terrestrial biodiversity conservation.

Secondly, based on water pollution control and riverine habitat restoration, a biomanipulation approach could be applied to promote the ecological restoration of the regional freshwater ecosystem in accordance with ecological principles. Aquatic and aquatic–terrestrial species (e.g., microbes, aquatic plants, macroinvertebrates) are basic components of freshwater food webs; they play essential roles in nutrient cycling, metabolic processes, and many other biogeochemical processes and ecosystem functions [94,95]. Hence, promoting the protection and reproduction of biological resources of instream and riparian zone systems, is substantial for increasing the regional biodiversity, and thus enhancing the ecosystem functioning, services, and ecological resilience [96,97]. Riverine biodiversity could be restored through diverse ecological engineering approaches or technologies, for instance by introduction of native species, regulation and management of aquatic organisms, etc.

### 4.3. Ecological Network

#### 4.3.1. Characteristic of the Ecological Network

Under climate change and anthropogenic disturbance, freshwater ecosystems are highly threatened by habitat fragmentation [98]. Due to disconnection of regional patches and limited migration opportunities in stream networks, habitat fragmentation becomes one of the main drivers of biodiversity loss and the diminishing of ecological security patterns [99]. The Suzhou Grand Canal was also suffering from habitat fragmentation to some extent, especially in the upstream canal, the habitat connectivity in all study regions was relatively low (no more than 11%). As a result of habitat loss and fragmentation, the drainage complexity and structural stability of Suzhou watershed had declined by 14.97% and 7.87%, respectively, from the 1980s to the 2010s [100]. The high-intensity anthropogenic disturbance has seriously influenced the habitat connectivity, the ecosystem function, as well as the ecological balance of freshwater ecosystems in Suzhou. While continuous land use changes disconnected the natural habitat (e.g., the linkage between the river and adjacent wetland ecosystem), leading to habitat fragmentation and reduced living capacity of aquatic biota, non-point source pollution discharged from adjacent industries and residential areas contaminated the instream and riparian area, aggravating the decline of biodiversity, ecosystem function, and ecological stability of the freshwater ecosystem. Although 142 km of landscape projects implemented in recent years has increased 2 million square meters of green space along the canal, the ecological network has not been fully established within the canal region.

Habitat loss and fragmentation could be attributed either to natural disturbance (e.g., climate change), or anthropogenic disturbance, by clearing-cutting of forest, cultivation of grasslands and croplands, construction of dams and reservoirs, and development of urban manufacturers. On the other hand, human-induced fragmentation tends to occur more rapidly than natural fragmentation [33]. Although no dams or reservoirs were constructed in the study region, the establishment of heavy pollution industries, extensively managed agricultural lands, and the development of urban systems, all account for the fragmentation of the living habitat, for aquatic or terrestrial communities. Whereas, due to the limited availability of socioeconomic data, the driving factors selected in this article do not include all the influencing factors. Ecosystem-related socioeconomic status, as well as biodiversity and ecosystem function conditions in the river and riparian corridor, can be included as alternative factors for future research.

#### 4.3.2. Recommendations for Ecological Network Construction

Landscape connectivity is vital for enhancing the movement of aquatic–terrestrial species across the landscape, and thus maintaining genetic diversity under climatic or land-use changes [99]. It is important to connect ecological corridors and improve the regional ecological network. Firstly, ecological resources (e.g., grasslands, forest lands, and wetlands) form the basic components of the riverine ecological network [101]. Nevertheless, the research results demonstrated a declined habitat quality and habitat connectivity of the canal ecosystem. Hence, it would be favorable for ecological planners to explore the key regional ecological nodes within the river corridor, to restore the heterogenous habitat of the ecological nodes and diverse ecological resources, with a coordinated management standard; next, they could restore the well-designed ecological corridors through the linkage of these ecological nodes with various ecological resources (etc., lakes, tributaries, and canal parks), distributed within the canal corridor area.

Secondly, ecological network construction is vital for connecting fragmented habitats, enhancing the connectivity of regional landscapes, facilitating the movement of aquatic–terrestrial species across the landscape, and thereby improving the ecosystem stability and regional ecological security [102]. Considering the high habitat fragmentation of the studied canal region, it is necessary to build a systematic ecological network through the development of solid linkages among diverse ecological systems. Horizontally, connecting the blue–green system, including the canal, riverside buffer zone, and local resources (etc., adjacent residential or industrial areas, lakes or forest lands) within the study region, would assist the rebuilding of continuous and smooth ecological corridors with distinguished local features; vertically, identifying the cross-regional ecological corridor nodes, and rebuilding the potential structural and functional linkages between these nodes and ecological resources of different regions, would form ecological corridors from the north to the south canal. Finally, a blue–green interweaving ecological network could be systematically established at the city-level, by horizontal and vertical interconnection of diverse lines and patches.

## 5. Conclusions

Based on LULC classification, the InVEST habitat quality model, FRAGSTATS 4.2 landscape program, and field investment, this study takes the Suzhou Grand Canal as a case study to assess the land use characteristics, habitat features, riparian vegetation, and water quality, of a freshwater ecosystem. Above all, this research illustrated a simplified habitat composition, a relatively low habitat quality and habitat connectivity of the canal corridor, a relatively high habitat fragmentation, and a greater nutrient and COD level in the surface water—especially in the upstream and midstream canal, which indicated that the canal ecosystem was moderately degraded. Multiple stressors such as residential discharge, runoff from the established heavy pollution industry, extensively managed agriculture, a developed urban system, and tail water from wastewater treatment plants, all accounted for the eutrophication of the canal. Additionally, urbanization-induced land use changes mainly promoted the habitat fragmentation/degeneration for aquatic or terrestrial communities. To summarize, all urbanization-induced land use change and related stressors had a synergistic contribution to the deterioration of the canal ecosystem.

For the comprehensive restoration of the freshwater ecosystem, creating a systematic connected ecological corridor was highly recommended. Practically, strengthening the water pollution control from the source would stimulate the protection of the water resource; restoring wetlands linking rivers and lakes collaboratively within the watershed network, would promote the survival and reproduction of aquatic and terrestrial species; constructing a comprehensively linked watershed ecological network by connecting the horizontal and vertical ecological corridors, would enhance the movement of aquatic–terrestrial species, the maintenance of the genetic diversity, and the resilience of watershed toward future climate change and anthropogenic disturbance. The results of this study provide a scientific basis for the protection of the canal ecosystem. The systematic ecological corridor restoration strategy developed in this study, provides guidelines on ecological corridor restoration and management of the freshwater ecosystem, which could be applied in other regions worldwide to: improve structure and function of watershed ecosystem; to enhance ecosystem service and ecological security for human beings; and to mitigate the impact of urbanization on the ecological environment, so as to realize the sustainable development of complex urban systems.

## Figures and Tables

**Table 1:** Maximum distance, impact weight and type of decay.

Threat	Max_Dist (km)	Weight	Decay
Cropland	0.5	0.5	exponential
Built area	12	1.0	linear

**Table 2:** Species’ habitat preference and threat sensitivity.

Name	Habitat	Cropland	Built Area
Wetland	1.0	0.7	0.9
Forest land	1.0	0.6	0.9
Grassland	0.6	0.5	0.6
Cropland	0.4	0.3	0.5
Built area	0	0	0
Bare ground	0	0	0

**Table 3:** Numbers of manufacturing industries, agricultural companies, and Sewage Treatment Plants surrounded by the Suzhou Grand Canal.

Area	Chemical Enterprise	Textile Enterprise	Papermaking Enterprise	Concrete Enterprise	Metallurgical Enterprise	Agricultural Business	Sewage Treatment Plant
Upstream	26	17	15	44	21	6	3
Midstream	29	55	8	31	8	6	2
Downstream	30	2321	48	83	57	9	15
Total	63	2562	71	158	86	21	20

**Table 4:** Habitat metrics including mean habitat quality, mean habitat degradation, mean habitat connectivity and fragmentation, and vegetation coverage of the study area within the Suzhou Grand Canal in 2021.

Area	Mean Habitat Quality	Mean Habitat Degradation	Mean Habitat Connectivity (%)	Mean Habitat Fragmentation	Vegetation Coverage (%)
Upstream	0.110	0.070	8.673	0.154	35.929
Midstream	0.109	0.094	10.980	0.105	30.719
Downstream	0.282	0.071	4.585	0.037	41.029

**Table 5:** Data of physico-chemical parameters, including pH, dissolved oxygen (DO), ammonium (NH_4_-N), nitrate (NO_3_-N), total nitrogen (TN), total phosphorus (TP), chemical oxygen demand (COD), and conductivity in five sections of the Suzhou Grand Canal, and the difference of physico-chemical indexes between each section of the canal within Suzhou City Region, PRC. Mean values (±SE, *n* = 3) are presented for physico-chemical parameters. Asterisks are significant level at *p* < 0.05, two asterisks are significant level at *p* < 0.01.

Section	pH		DO (mg/L)		NH_4_-H (mg/L)		NO_3_-N (mg/L)		TN (mg/L)		TP (mg/L)		COD (mg/L)		Conductivity	
A	6.950 ± 0.071		7.700 ± 1.556		0.637 ± 0.042		0.630 ± 0.205		1.200 ± 0.184		0.137 ± 0.000		24.500 ± 3.536		396.500 ± 33.234	
B	6.900 ± 0.000		8.233 ± 0.115		0.617 ± 0.040		1.167 ± 0.117		1.540 ± 0.137		0.191 ± 0.020		26.000 ± 1.000		436 ± 28.478	
C	6.900 ± 0.000		8.600 ± 0.141		1.053 ± 0.209		0.723 ± 0.161		1.905 ± 0.022		0.233 ± 0.010		32.500 ± 4.950		421.500 ± 28.991	
D	7.000 ± 0.000		2.235 ± 0.276		0.671 ± 0.169		5.000 ± 0.849		2.555 ± 0.092		0.206 ± 0.144		39.00 ± 16.971		610 ± 15.556	
E	7.000 ± 0.000		0.631 ± 0.269		2.143 ± 0.150		6.067 ± 0.379		2.357 ± 0.427		0.200 ± 0.085		60.667 ± 9.609		617.667 ± 60.053	
	**pH**		**DO**		**NH_4_-N**		**NO_3_-N**		**TN**		**TP**		**COD**		**Conductivity**	
	Difference	*p*	Difference	*p*	Difference	*p*	Difference	*p*	Difference	*p*	Difference	*p*	Difference	*p*	Difference	*p*
AB	0.05	0.335	−0.533	0.853	0.023	0.9	−0.532	0.604	−0.34	0.6	−0.053	0.9	−1.5	0.9	−39.5	0.789
AC	0.05	0.41	−0.9	0.597	−0.415	0.237	−0.09	0.9	−0.705	0.134	−0.095	0.684	−8	0.866	−25	0.9
AD	−0.05	0.41	5.465	0.001 **	−0.03	0.9	−4.365	0.001 **	−1.355	0.006 **	−0.065	0.887	−14.5	0.492	−213.5	0.006 **
AE	−0.05	0.335	5.557	0.001 **	0.01	0.9	−5.432	0.001 **	−1.157	0.009 **	−0.06	0.882	−36.167	0.014 *	−221.167	0.003 **
BC	0	0.9	−0.367	0.9	−0.438	0.15	0.442	0.726	−0.365	0.546	−0.042	0.9	−6.5	0.9	14.5	0.9
BD	−0.1	0.027 *	5.998	0.001 **	−0.053	0.9	−3.833	0.001 **	−1.015	0.019 *	−0.012	0.9	−13	0.506	−174	0.012 *
BE	−0.1	0.015 *	6.09	0.001 **	−0.013	0.9	−4.9	0.001 **	−0.817	0.031 *	−0.007	0.9	−34.667	0.010 **	−181.667	0.005 **
CD	−0.1	0.041 *	6.365	0.001 **	0.385	0.29	−4.275	0.001 **	−0.65	0.177	0.03	0.9	−6.5	0.9	−188.5	0.013 *
CE	−0.1	0.027 *	6.457	0.001 **	0.425	0.166	−5.342	0.001 **	−0.452	0.371	0.035	0.9	−28.167	0.048 *	−196.167	0.006 **
DE	0	0.9	0.092	0.9	0.04	0.9	−1.067	0.111	0.198	0.9	0.005	0.9	−21.667	0.137	−7.667	0.9

## Data Availability

The data presented in this study are available on request from the corresponding author.

## Appendix A

**Table A1:** Standard limit of basic item—Surface Water Environmental Quality Standard GB3838-2002.

Variables		I	II	III	IV	V
pH		6~9				
DO (mg/L)	≥	7.5	6	5	3	2
COD (mg/L)	≤	15	15	20	30	40
NH_4_-N (mg/L)	≤	0.15	0.5	1.0	1.5	2.0
TN (mg/L)	≤	0.2	0.5	1.0	1.5	2.0
TP (mg/L)	≤	0.02	0.1	0.2	0.3	0.4
BOD (mg/L)	≤	3	3	4	6	10

**Table A2:** Criteria and Grading Methods for Nutritional Status Evaluation in Technical Regulations for Quality Evaluation of Surface Water Resources SL 395-2007.

Nutrient Status *EI* = Nutritional Index	Evaluation Value (*En*)	Total Nitrogen (TN mg/L)	Total Phosphorus (TP mg/L)
Oligotropher 0 ≤ EI ≤ 20	10	0.020	0.001
	20	0.050	0.004
Mesotropher 20 < EI ≤ 50	30	0.100	0.010
	40	0.300	0.025
	50	0.500	0.050
Light eutropher 50 < EI ≤ 60	60	1.000	0.100
Middle eutropher 60 < EI ≤ 80	70	2.000	0.200
	80	6.000	0.600
Hyper eutropher 80 < EI ≤ 100	90	9.000	0.900
	100	16.000	1.300
